# Auto-Segmentation and Auto-Planning in Automated Radiotherapy for Prostate Cancer

**DOI:** 10.3390/bioengineering12060620

**Published:** 2025-06-06

**Authors:** Sijuan Huang, Jingheng Wu, Xi Lin, Guangyu Wang, Ting Song, Li Chen, Lecheng Jia, Qian Cao, Ruiqi Liu, Yang Liu, Xin Yang, Xiaoyan Huang, Liru He

**Affiliations:** 1 Sun Yat-sen University Cancer Center, Guangzhou 510060, China; huangsj@sysucc.org.cn (S.H.);; 2 United Laboratory of Frontier Radiotherapy Technology of Sun Yat-sen University & Chinese Academy of Sciences Ion Medical Technology Co., Ltd., Guangzhou 510060, China; 3 School of Biomedical Engineering, Southern Medical University, Guangzhou 510515, China; 4 Department of Radiation Oncology, Sun Yat-sen Memorial Hospital, Guangzhou 510012, China; 5 Shanghai United Imaging Healthcare Co., Ltd., Shanghai 201807, China

**Keywords:** auto-segmentation, auto-planning, automated radiotherapy, prostate cancer

## Abstract

**Objective:** The objective of this study was to develop and assess the clinical feasibility of auto-segmentation and auto-planning methodologies for automated radiotherapy in prostate cancer. **Methods**: A total of 166 patients were used to train a 3D Unet model for segmentation of the gross tumor volume (GTV), clinical tumor volume (CTV), nodal CTV (CTVnd), and organs at risk (OARs). Performance was assessed by the Dice similarity coefficient (*DSC*), the *Recall*, *Precision*, *Volume Ratio (VR)*, the 95% Hausdorff distance (*HD95*%), and the volumetric revision degree (*VRD*). An auto-planning network based on a 3D Unet was trained on 77 treatment plans derived from the 166 patients. Dosimetric differences and clinical acceptability of the auto-plans were studied. The effect of OAR editing on dosimetry was also evaluated. **Results**: On an independent set of 50 cases, the auto-segmentation process took 1 min 20 s per case. The *DSCs* for GTV, CTV, and CTVnd were 0.87, 0.88, and 0.82, respectively, with *VRD*s ranging from 0.09 to 0.14. The segmentation of OARs demonstrated high accuracy (*DSC* ≥ 0.83, *Recall/Precision* ≈ 1.0). The auto-planning process required 1–3 optimization iterations for 50%, 40%, and 10% of cases, respectively, and exhibited significant better conformity (*p* ≤ 0.01) and OAR sparing (*p* ≤ 0.03) while maintaining comparable target coverage. Only 6.7% of auto-plans were deemed unacceptable compared to 20% of manual plans, with 75% of auto-plans considered superior. Notably, the editing of OARs had no significant impact on doses. **Conclusions:** The accuracy of auto-segmentation is comparable to that of manual segmentation, and the auto-planning offers equivalent or better OAR protection, meeting the requirements of online automated radiotherapy and facilitating its clinical application.

## 1. Introduction

Prostate cancer is the most commonly diagnosed cancer in men, accounting for 29% of all cancer cases in the USA in 2024 [1], with its incidence rapidly increasing in China [2]. Over the past few decades, radiotherapy (RT) has been increasingly used for the curative treatment of prostate cancer; however, the potential for radio-toxicity has drawn increased attention. Since prostate RT typically takes several weeks, substantial changes in tumors and surrounding organs at risk (OARs) can occur between the initial planning and the delivery of fractional doses [3,4,5]. While image-guided radiotherapy (IGRT) can minimize the fractional variation, it cannot fully compensate for tumor shrinkage and growth, or anatomical variations like rectal filling or organ deformation [6]. These variations might result in unintended doses to both the targets and OARs, leading to treatment interruption or the need for re-planning.

Adaptive radiotherapy (ART) can address inter-fraction variations by updating the treatment plan based on the updated image and contours during treatment [7]. There are two types in clinical practice: offline ART and online ART. Offline ART is usually performed periodically or when significant anatomical changes occur. Imaging, contouring, plan adaptation, and quality assurance (QA) are performed when the patient is not in the treatment room, often leading to treatment interruption. In contrast, online ART is performed immediately prior to treatment delivery when the patient is immobilized on the treatment couch, minimizing the time gap between plan adaptation and delivery and allowing more frequent adaptation to enhance treatment quality.

All-in-one (AIO) radiotherapy streamlines the RT process by integrating simulation, contouring, planning, and delivery in a single session, thereby eliminating waiting times between steps and preventing anatomical changes that occur over days or weeks. This approach has shown encouraging results in nasopharyngeal treatment in our center [8], significantly reducing the deviations of anatomical changes resulting from preparation time. Both AIO and online ART represent critical advances in radiotherapy, as they can improve local tumor control while reducing toxicity to OARs. For prostate cancer, ART has shown potential in reducing toxicity rates and improving target coverage [9,10,11]. However, it presents several challenges. The workflow comprises online imaging, segmentation, planning, plan approval, and QA [12]. Since the patient is on the couch during adaptation, the whole process should be completed within minutes to minimize the anatomical variations between plan creation and delivery. The time constraints, particularly for online segmentation and planning, are the focus of our study.

Recent advances in artificial intelligence (AI) have developed auto-segmentation techniques that achieve great accuracy in many anatomical sites [13,14]. While many studies on prostate cancer primarily focus on OARs and prostate/seminal vesicles [15], fewer studies address clinical target volumes (CTVs) and nodal CTVs (CTVnds). Additionally, we provided the most comprehensive OAR segmentation, including the bladder, rectum, small intestine, colon, anal tube, penile bulb, femoral heads, and pelvic bone, all of which are closely related to radiotherapy toxicity. For online plan adaptation, various AI-based tools, such as dose–volume histogram (DVH) predictors, have emerged [16]. The DVH predictor estimates the optimal DVHs as a reference for plan evaluation. These studies have demonstrated good performance in geometric metrics, such as the Dice similarity coefficient (*DSC*), the Hausdorff distance (*HD*), the dosimetric difference, etc. However, researchers and clinicians have claimed that these metrics do not fully represent the clinical acceptability of a structure or a plan [17,18]. Therefore, it is essential to evaluate the performance of auto-segmentation and auto-planning in clinical practice.

While individual components of automated workflows have been investigated, few studies have evaluated an integrated approach combining both auto-segmentation and auto-planning specifically for prostate cancer. This integration is crucial, as errors or inefficiencies in either component can render the entire automated workflow impractical for routine clinical use. A CT-integrated linac named uRT-linac 506C (United Imaging Healthcare [UIH] Co., Shanghai, China) at our center [19] provides an ideal platform for implementing and validating such a comprehensive automated solution. It combines a 16-slice helical CT scanner, facilitating both simulation and planning. The treatment planning system (TPS) offers customized auto-segmentation and planning, streamlining the entire automated RT, AIO [20], and online ART [21], along with the delivery system.

This study aimed to evaluate the clinical performance of a CT-based deep learning auto-segmentation for prostate targets (gross tumor volume (GTV), CTV, CTVnd) and OARs, as well as auto-planning for automated RT for definite prostate cancer. We assessed the geometric performance and modification degree of the auto-segmented contours. For auto-planning, dosimetric difference and blind evaluation were conducted. Finally, the dosimetric difference of auto-plans generated with edited versus unedited OARs was analyzed to investigate the impact of organ editing on treatment outcome.

## 2. Materials and Methods

### 2.1. Imaging Data and Manual Delineation

A dataset of 166 prostate patients treated with definite radiotherapy at our institution between 2020 and 2024 was retrospectively selected for model development. An additional 50 patients were used as an independent testing set. All patients were scanned on a Big Bore CT scanner (Brilliance™CT, Philips, Eindhoven, The Netherlands) following an in-house prostate protocol [22]. The scanning parameters included a voltage of 140 kV, 300 mAs, with both scanning and reconstruction slice thickness set to 3 mm. The CT images (paired non-contrast CT and contrast-enhanced CT) were transferred to the Monaco workstation (V5.11.03, Elekta, Stockholm, Sweden). Targets and OARs were contoured according to the recommendations of the Radiation Therapy Oncology Group (RTOG). The GTV comprised the prostate alone for low-risk patients and the prostate and seminal vesicles for intermediate- or high-risk patients. The CTV included the subclinical regions of the GTV, such as the extent of invasion in the prostate or seminal vesicles. CTVnd included the pelvic lymph nodes. CTVs were expanded by approximately 5–7 mm (3–5 mm posteriorly) to produce planning tumor volumes (PTVs). The prescription doses were 67.5 Gy for GTV/PGTV, 65 Gy for CTV/PCTV, and 45 Gy for CTVnd/PCTVnd, delivered in 25 fractions. The bladder was fully contoured with the bladder wall and entire cavity. The rectum was contoured from the rectosigmoid junction downward to the anorectal junction, encompassing the rectal wall, with the anal canal typically delineated from the dentate line to the anus. The small intestine was identified by locating tortuous pelvic loops, which were contoured based on bowel wall thickness and the presence of intraluminal gas/fluid. The colon was contoured from the sigmoid origin to the recto-sigmoid junction. The penile bulb was contoured by locating the oval soft tissue density below the pubic symphysis. The pelvic bone was contoured with the cortex and medullary cavity. Lastly, the left and right femoral heads were fully outlined. Each patient was treated with a 6 MV linear accelerator with a volumetric modulated arc therapy (VMAT) plan. Plan optimization was performed on constraints for PTVs and OARs based on QUANTEC data and our center’s specific requirements (details presented in Table 1). This study was approved by the Institutional Ethics Committee (NO. SL-G2023-209-1).

### 2.2. Model Development

#### 2.2.1. Auto-Segmentation Model

To achieve highly precise segmentation results, we introduced a refined 3D Unet [23]. This network boasts a U-shaped architecture comprising encoding and decoding parts, integrated with 3D convolutional kernels. The utilization of 3D convolutional kernels is prevalent in extracting richer contextual information from 3D medical images.

**Figure 1:**
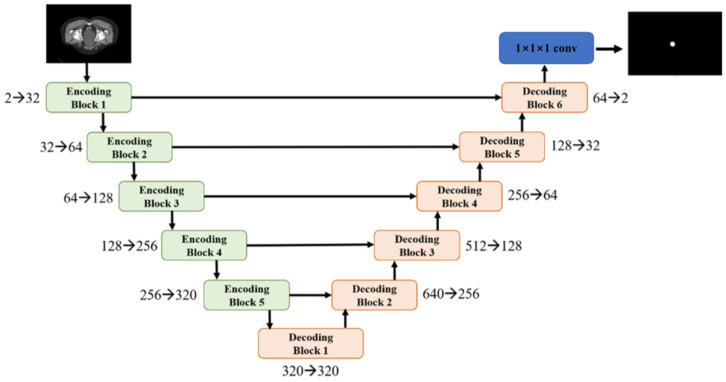
The structure of segmentation model.

As depicted in Figure 1, the encoder comprises five blocks, each constructed with two basic 3D convolutional blocks. Analogous structures are employed in the decoder part, with each decoding block containing two 3D convolutional blocks followed by a transposed convolutional block. However, the final decoding block (depicted in Figure 1) exhibits a slight deviation, as its transposed convolutional block is substituted with a 1 × 1 × 1 convolutional block to yield a two-channel output, representing the segmentation and background probabilities.

The final segmentation results are then derived through the segmentation layer. The numbers accompanied by arrows indicate the channel expansion in the encoder part and channel reduction in the decoder part, highlighting the transformation of feature maps throughout the network.

Our model was implemented using PyTorch 1.6 [24] and executed on an Ubuntu platform equipped with an Nvidia RTX 3090 GPU. The training strategy closely resembles nnUnet [25], a self-configuring deep learning framework designed for biomedical image segmentation. nnUnet automates hyperparameter tuning and data preprocessing, enhancing generalization performance across diverse datasets. During the training phase, we set the initial learning rate to 0.001 and conducted a total of 300 training epochs. Prior to feeding the data into the network, we enhanced the training set through techniques such as random flipping, cropping, scaling, and rotations to augment the dataset.

The retrospectively collected 166 patients were randomly separated into a training set with 132 patients and a test set with 34 patients. Total time costs were around 12 h for each target and OARs training process. To enhance efficiency and accuracy, the OARs were trained in a single process, while targets were trained individually.

#### 2.2.2. Auto-Planning Model

**Figure 2:**
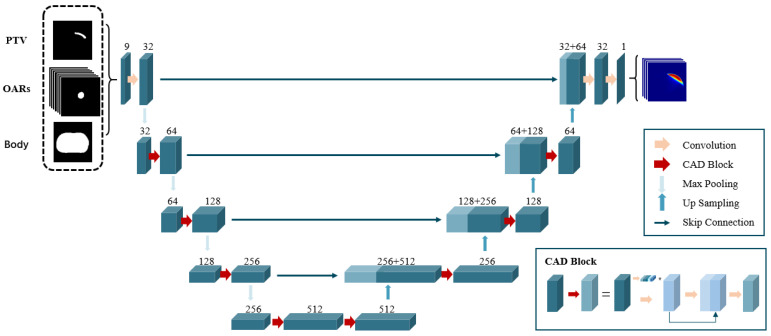
Dose prediction model based on 3D-UNet-based neural network mode.

Data from 77 definitive prostate cancer patients treated at our center were retrospectively collected. From these cases, we selected high-quality treatment plans that met strict clinical criteria, including optimal target coverage and OAR sparing, approved by senior radiation oncologists with minimal modifications. The final dataset comprised 67 cases with excellent plan quality for model training and 10 cases for testing. This selection strategy ensured that the model learned from optimal treatment plans, improving the quality of the automated planning. For training, we used the selected plans and extracted the PTVs and OARs as input. The dose distribution within the patient’s body served as the ground truth. The 3D-UNet-based neural network model predicts 3D dose distributions directly [26]. This architecture extends the well-known UNet structure to three dimensions, enabling it to process volumetric medical images effectively. As shown in Figure 2, the model consists of an encoder on the left and a decoder on the right. Both components employ modules with convolutional layers and attention mechanisms (CAD Blocks). Each CAD Block begins with a channel attention module to assign adaptive weights to different channels to emphasize more important features. This is followed by a cascaded convolution that expands the channel dimension to enhance feature extraction. A final reduction convolution maintains computational efficiency by reducing channels to the target output size.

During the encoding process, the number of convolutional filters in each module is 32, 64, 128, 256, and 512, respectively. A Max Pooling layer reduces the feature map size by half after each encoding step. In the decoding phase, upsampling doubles the feature map size. Except for final output layer, which uses 1 × 1 × 1 convolutional filters, all other convolutional filters use 3 × 3 × 3 filters. A skip connection mechanism links the encoder and decoder to fuse shallow image information with deep semantic features. The model incorporates GroupNorm layers instead of BatchNorm, dividing channels into groups and normalizing within each group, which enhances training stability across varying batch sizes. For weight initialization, we employed the He_normal method (also known as Kaiming initialization), which initializes weights from a truncated normal distribution centered on 0 with a standard deviation $\sqrt{2/\mathrm{fan\_in}}$, where fan_in is the number of input units in the weight tensor. This initialization approach is particularly effective for models using ReLU activations, as it helps maintain proper activation and prevents vanishing or exploding gradients during training. The techniques collectively enhance the model’s generalization capabilities.

The input of the model was 3D contouring data, and the output was the predicted 3D dose distribution. The models were trained using the Adam optimizer [27], which was selected after preliminary tests showed it provided more stable convergence and slightly better performance compared to other optimizers, including AdamW, despite the latter’s decoupled weight decay. The initial learning rate was set to 1 × 10^−4^, with a total of 1000 training epochs, and a weight decay coefficient of 1 × 10^−4^. Mean Squared Error (MSE) was chosen as the loss function. Python 3.8 was utilized as the programming language, and Pytorch 1.13 was selected as the deep learning framework. The training process was executed on an NVIDIA RTX 3090 GPU running on a Linux system.

The automated planning framework integrates several key components to generate clinically acceptable plans with minimal user intervention. The framework relies on two main inputs: 3D dose distribution predicted by neural network model, and a clinical goal sheet. The clinical goal sheet is derived from radiologists’ prescribed wish-list, including target prescription and dosimetric requirements for organs of interest. The clinical goal sheet allows prioritization, enabling the representation of trade-offs between organs and targets, as well as defining the importance of different organs.

During optimization, the AutoPlan engine maps each prioritized goal to an optimization objective. It adjusts the goal dose or volume based on the predicted 3D dose distribution, then performs multiple optimization loops to iteratively adjust dosimetric parameters to meet the goals. Several auxiliary optimization objectives are added in, including targets for internal dose control and dose drop-off outside targets. Different optimization strategies are employed in each loop, such as R50% control (which limits the ratio of the volume receiving 50% of the prescription dose to PTVs, thereby controlling dose spillage to OARs), target dose conformity optimization, global max dose control, and prioritization-based optimization. All automated plans are generated using coplanar 6 MV photon beams within the UIH TPS, and dose distributions are calculated using the GPU-based Monte-Carlo dose engine.

### 2.3. Geometric Evaluation

The geometric similarity between two different contours is determined using metrics: *DSC*, *Recall*, *Precision*, *Volume Ratio* (*VR*), *95% undirected HD* (*HD95%*).

The *DSC* is given as

(1) $$\mathrm{DSC}=\frac{2\left|x\cap y\right|}{\left|x\right|+\left|y\right|}$$

where x and y are the manual and auto-segmentation contours, respectively.

The *Recall*, or true positive rate, is given as

(2) $$\mathrm{Recall}=\frac{\left|x\cap y\right|}{\left|x\right|}$$

The *Precision*, or positive predictive value, is given as

(3) $$\mathrm{Precison}=\frac{\left|x\cap y\right|}{\left|y\right|}$$

The 95th directed percent Hausdorff measure is the 95th-percentile distance over all distances from points in x to their closet point y, and $d\left(x,y\right)$ is the distance between point $x_{i}$ in x and point $y_{i}$ in y. Denoting the 95th percentile as *K_95_*, this is given as:

(4) $${\vec{d}}_{H,95}\left(x,y\right)=K_{95}\left(\min_{y\in \left|y\right|}d\left(x,y\right)\right)\forall_{x_{i}}\in x$$

The *HD95%* is defined as the average of the two directed distances:

(5) $$\mathrm{HD}95\%=\frac{{\vec{d}}_{H,95}\left(x,y\right)+{\vec{d}}_{H,95}\left(y,x\right)}{2}$$

In addition, volumetric revision degree (*VRD*) was defined as the volume required to be edited divided by the volume of segmented contours [28]. It was classified into 4 levels: no revision (*VRD* = 0), minor revisions (0 < *VRD* ≤ 0.1), moderate revisions (0.1 < *VRD* < 0.2), and major revisions (*VRD* ≥ 0.2).

### 2.4. Dosimetric Evaluation of Automated Prostate Treatment Planning

Dosimetric differences between manual plans and auto-plans were compared for both targets and OARs, including the homogeneity index (*HI*), conformity index (*CI*), maximum dose of target, target coverage, and the DVH. According to the International Commission on Radiation Units report No. 83 [29], the *HI* and *CI* were defined in Equations (6) and (7), respectively:

(6) $$\mathrm{HI}=\frac{D_{2\%}-D_{98\%}}{D_{50\%}}$$

(7) $$\mathrm{CI}=\frac{{\left({\mathrm{TV}}_{\mathrm{Target}100\%}\right)}^{2}}{\mathrm{TV}\times V_{\mathrm{Target}100\%}}$$

*D_2%_*, *D_98%_*, and *D_50%_* are the doses received by *2%*, *98%*, and *50%* of targets, respectively. *CI* values approaching 1 reflect improved conformity to the target. *TV* is the volume of the target; *TV_Target100%_* is the target volume covered by the prescription dose, and *V_Target100%_* represents the total volume covered by the prescription dose. The smaller the *HI*, the more uniform the target dose.

A clinical evaluation was conducted to assess the feasibility of automated planning. In total, 40 plans (20 manual plans and 20 auto-plans) for 20 patients were reviewed by 3 radiologists. These 20 patients were randomly selected from the independent 50 cases that were optimized only once. Radiologists reviewed all the plans and determined whether the plan could be approved for clinical treatment, and subsequently chose the better plan between the manual plans and auto-plans. In addition, each radiologist scored each plan using a 5-point Likert scale.

The 5-point scale was as follows:

1. Reject/Unacceptable, unusable: Plans of poor quality that cannot be used.
2. Unacceptable with major changes required: Edits are required to ensure appropriate treatment and are significant enough that the user would prefer to start from scratch.
3. Unacceptable with minor changes required: Clinically important edits for which it is more efficient to edit the plans than to start from scratch.
4. Acceptable: Stylistic differences, but not clinically important.
5. Perfect: Clinically acceptable, could be used for treatment without any changes.

### 2.5. Statistical Analysis

Pairwise comparisons of segmentation and planning between manual and auto were performed using SPSS version 22.0 (IBM Corp., Armonk, NY, USA). A paired *t*-test was used when the data was normally distributed, otherwise the Wilcoxon test was applied. *p* < 0.05 was considered statistically significant.

## 3. Results

### 3.1. Geometrical Analysis of Target and OARs

**Figure 3:**
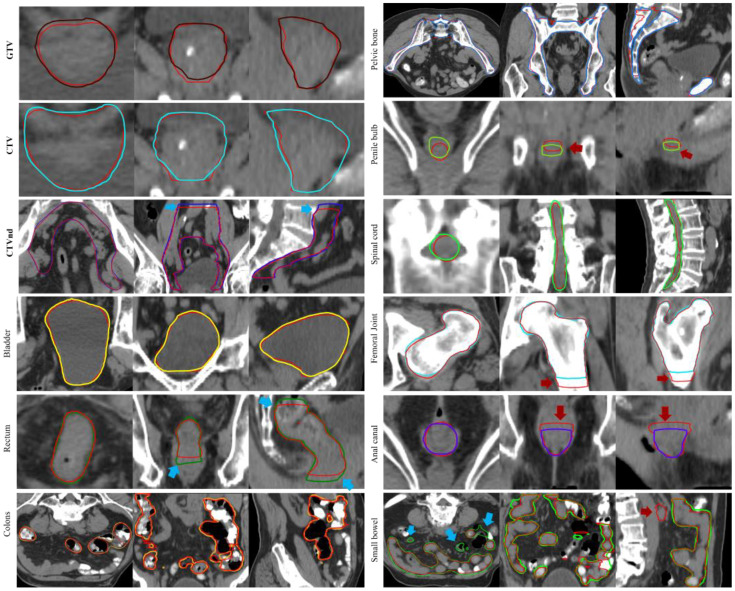
Comparative visualization of targets and OARs between auto-segmented and manual contours. Red lines represent manual contours, while other lines represent auto-segmented contours. The red arrows indicate areas where auto-segmentation is under-contoured, while the blue arrows indicate areas where auto-segmentation is over-contoured. GTV: gross tumor volume; CTV: clinical tumor volume.

Table 2 summarizes the geometric evaluation of the auto-segmented versus manual contours, with manual contours serving as the gold standard. For the targets, the mean *DSC*s of GTV, CTV, and CTVnd were 0.87, 0.88 and 0.82, respectively, and the corresponding *VRD*s were 0.09, 0.14, and 0.12, respectively. The *HD95%* (16.5 mm) of the CTVnd was 16.5 mm, which was greater than those for GTV and CTV (4.70 mm and 5.26 mm, respectively). The *Recall* and *Precision* of the targets were ≥0.89 and ≥0.77, respectively. For OARs, the bladder exhibited the best performance, with a mean *DSC* of 0.96, an *HD95%* of 3.35 mm, and a *VRD* of 0.03. The pelvic bone also showed excellent results, with a mean *DSC* of 0.91, an *HD95%* of 5.97 mm, and a *VRD* of 0.08. The penile bulb exhibited the lowest performance, with a mean *DSC* of 0.65, an *HD95%* of 3.73 mm, and a *VRD* of 0.40. The *Recall* and *Precision* of the OARs were close to 1.0, except for the penile bulb. These results indicate that the auto-segmented contours and manual contours had similar volumes. The averaged auto-segmentation time of all targets and OARs was 1 min 20 s. Details can be found in Table 2 and Figure 3.

### 3.2. Analysis of Auto-Plans and Manual Plans

#### 3.2.1. Dosimetric Analysis of Auto-Plans and Manual Plans with Manual Contours

The planning quality and efficiency of 50 patients were retrospectively studied. All plans were generated through automatic optimization by senior medical physicists using a unified set of constraints on the UIH TPS. Manual plans were those that had been approved for clinical treatment. When an initial automatic plan failed to meet the clinical requirements, priorities of the non-compliant organs were adjusted for one or more times until the plans met the clinical criteria. The percentages of one/two/three optimization attempts for planning approvals of the 50 automated plans were 50%, 40%, and 10%. The average duration for automatic planning was approximately 5 min 51 s.

Table 3 reports the dosimetric comparison of the PTVs. There was no significant difference in target coverage between auto-plans and manual plans (*p* ≥ 0.05). However, auto-plans exhibited better target conformity than manual plans (*p* ≤ 0.01). For the homogeneity of PCTV and PGTV, the manual plans showed slightly better performance than auto-plans. No significant difference was found in homogeneity of PCTVnd.

**Figure 4:**
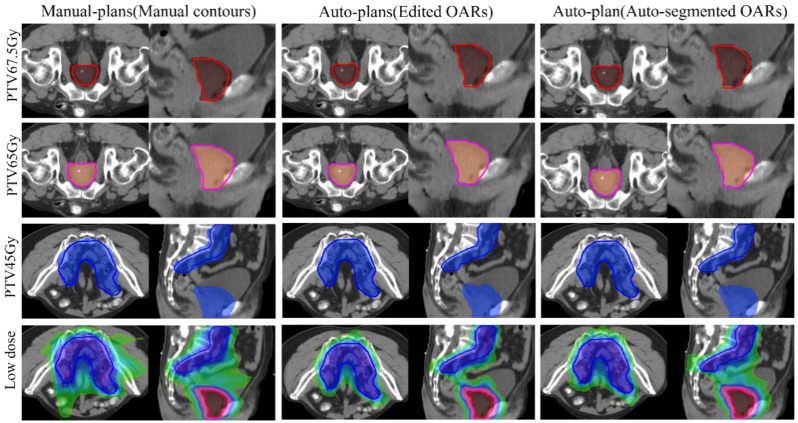
Dose distribution of manual plans (manual contours), auto-plans (edited OARs/manual OARs), and auto-plans (unedited OARs/auto-segmented OARs). Low dose refers to 30 Gy. The red, pink, and blue lines represent PGTV, PCTV, and PCTVnd, respectively. The shades of dark red, orange, blue, and green indicate dose ranges of 6750 cGy, 6500 cGy, 4500 cGy, and 3000 cGy.

In terms of OARs for prostate cancer, the auto-plans delivered lower doses to the rectum, bladder, spinal cord, and pelvic bone, except for the V65 Gy for the bladder and rectum (*p* ≥ 0.05). In addition, the V30 Gy for the small bowel and colon in the auto-plans was less than that of manual plans, as was the case with the anal canal V20 Gy. There was no significant difference for other dosimetric metrics; the details are displayed in Table 3 and Figure 4. In all, the auto-plans showed a better protection of OARs than manual plans with manual contours (contours were manually adjusted by doctors after auto-segmentation).

#### 3.2.2. Physician Evaluation of Auto-Plans and Manual Plans with Manual Contours

Plans of 20 patients were evaluated by three radiologists to determine if they complied with clinical standards. Two plans (auto-plan and manual plan) were evaluated for each patient. The results showed that 91.67% (55/60) of the manual plans and 96.67% (58/60) of the auto-plans could be approved for treatment by three radiologists, while the others were in need of revision and re-evaluation. Auto-plans were rated superior to manual plans in 75% (45 out of 60) of the reviews by the three radiologists.

**Figure 5:**
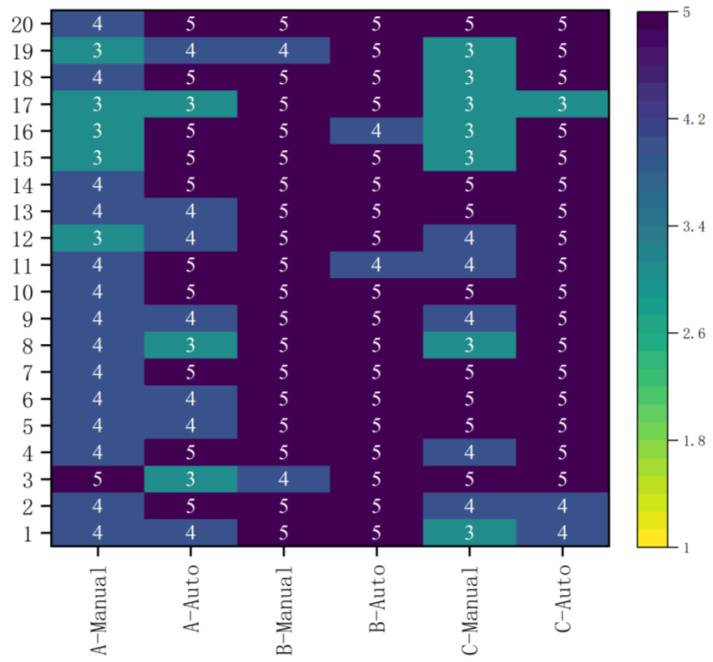
Five-point Likert scores for the auto-plans and manual plans by 3 radiologists (A, B, C).

According to the results of a 5-point Likert scale, 45% (27/60), 35% (21/60), and 20% (12/60) of the manual plans were scored as “perfect”, “acceptable”, and “unacceptable”, respectively. On the other hand, 75% (45/60), 18.33% (11/60), and 6.67% (4/60) of the auto-plans were scored as “perfect”, “acceptable”, and “unacceptable”, respectively. Details can be found in Figure 5.

### 3.3. Planning Comparison of Auto-Plans on Edited/Manual OARs and Unedited/Auto-Segmented OARs

To evaluate the effect of unedited contours on auto-planning during online ART, the dosimetric differences between auto-plans with edited/manual OARs and those with unedited OARs/auto-segmented OARs were investigated. Twenty automated plans with only one optimization were selected. The average planning time for one optimization of a plan was approximately 3 min 42 s. For PTVs, no significant difference was found for targets and OARs; details can be found in Table 3.

## 4. Discussion

With the integration of AI and radiotherapy, numerous novel radiotherapy techniques have emerged, such as AIO radiotherapy, on-line ART, etc. The application of these technologies in clinical practice is closely linked to the accuracy and efficiency of AI technologies. Many studies have focused on the accuracy of the AI network/algorithm, but quantitative assessment is not fully representative of clinical applicability. Hence, we quantitatively and qualitatively investigated the accuracy and efficiency of auto-segmentation and auto-planning in prostate automated RT workflow, e.g., one-shot RT and on-line ART.

Recent studies have demonstrated an excellent performance achieved by AI-based auto-segmentation tools for the prostate region. Wong et al. reported *DSC* values of 0.91, 0.97, 0.83, and 0.68 for femoral heads, bladder, rectum, and seminal vesicles, respectively [30,31,32]. Carina’s studies achieved slightly better performance for the femoral heads and seminal vesicles, with *DSC* values of 0.96 and 0.72, respectively; slightly worse performance for the bladder with a *DSC* value of 0.93; and similar performance for the rectum with a *DSC* value of 0.85 [30]. Overall, the segmentation performance for OARs is good and greatly reduces the time of the physician’s contouring. The target for prostate cancer patients, as the treatment target for GTV, CTV, and CTVnd, is the most important structure to contour. In clinical practice, it must be contoured by an experienced physician or oncologist. Carina’s team achieved a *DSC* value of 0.83 ± 0.05 for the prostate. Ghavami et al. reported a *DSC* value of 0.89 ± 0.03 for prostate segmentation based on 232 patients’ magnetic resonance (MR)-imaging data with five-fold cross-validation [33].

In our study, we present several innovations in both methodology and clinical application. Unlike most previous studies that focus solely on OARs or partial targets, our approach provides a comprehensive solution for the entire prostate radiotherapy workflow, simultaneously addressing targets (GTV, CTV, CTVnd) and multiple OAR segmentations with a refined 3D-UNet architecture, which is the most comprehensive segmentation for prostate cancer in recent studies. The integration of channel attention mechanisms in our CAD Blocks significantly enhances feature extraction and model performance. The *DSC*s for GTV, CTV, and CTVnd were 0.87 ± 0.06, 0.88 ± 0.12, and 0.82 ± 0.06, respectively, and the mean *VRD*s were 0.09, 0.14, and 0.12, respectively. This means that target edits require only minor revisions (0 < *VRD* ≤ 0.10) or moderate revisions (0.10 < *VRD* < 0.20). Notably, the deviations were primarily attributed to inconsistency in the superior–inferior directions, especially for CTVnd, as illustrated in Figure 3. The CTVnd included the range of the pelvic drainage area, and it is directly related to the stage of prostate patients. For OARs, the *DSC* was more than 0.83, except for the penile bulb. The *VRD* of OARs was close to 0.15, except for the penile bulb (0.40) and spinal cord (0.33), indicating minor revisions or moderate revisions. In addition, the auto-segmentation of targets and OARs can be performed in an average time of 1 min 20 s. The auto-segmentation network has been used in clinic practice and significantly reduced the revised time to about 2 min, which is comparable to current online adaptive reports [34,35,36,37]. Given the increasing use of MRI in prostate radiotherapy planning due to its superior soft tissue contrast, future research should focus on auto-segmentation in MRI. This study proved that the unified 3D Unet is effective for OARs and targets in CT images; we believe it is also suitable for MRI segmentation.

Online ART needs to finish the segmentation, planning, and treatment in minutes while the patients are on the treatment couch [38,39,40]. Consequently, rapid planning is essential. Many AI-based tools have enabled rapid planning with good performance [41,42], and automatic planning generally improves efficiency while reducing variability in plan quality. Our auto-planning approach introduces a novel two-stage workflow that combines deep learning dose prediction with multi-objective optimization, guided by a prioritized clinical goal sheet. This approach maintains equivalent target coverage while achieving superior conformity and OAR sparing, particularly for the bladder, rectum, pelvic bone, spinal cord, small-bowel, colon V30 Gy, and anal canal V20 Gy, compared to manual plans. These results are in line with the observed benefits of auto-planning in a previous study [43]. Meanwhile, the average time for one automatic planning is approximately 5 min and 51 s, significantly shorter than the time of manual planning (about 22 h). Our ongoing improvements in optimization algorithms and hardware configurations have demonstrated potential to further reduce this time to less than 3 min, making it even more suitable for time-sensitive clinical workflows such as one-shot RT and online ART. This significant time reduction represents a critical advancement for practical implementation in routine clinical practice.

In addition to a dosimetric investigation, we conducted a clinician evaluation to ensure the clinical applicability of the auto-plans. Based on the evaluations of three radiologists, auto-plans were deemed permissible for clinical use, and 75% of the automatic plans were considered superior to manual plans. While some auto-plans have shortcomings, such as max doses in OARs exceeding dose limits, the dose distribution of auto-plans was generally lower than that of manual plans. This was deemed acceptable for clinical practice by physicists and radiologists. Moreover, it was found that five manual plans, which were treatment plans, needed to be revised and re-evaluated due to insufficient plan conformity. These findings suggest that the auto-plans can serve as QA tools for planning in radiotherapy workflow and are essential for risk management in radiotherapy [44,45].

The limited amount of time remains a primary challenge for both one-shot RT and online ART. According to clinical experience, junior early-career doctors still need about 10 min to modify the auto-segmented organs as pelvic organs are particularly complex. Eliminating the need for expert contour modifications would improve the efficiency of the automatic workflow and facilitate broader implementation. Therefore, we investigated the dosimetric differences of plans based on edited OARs and unedited OARs. It was revealed that no difference was found in target coverage and OARs. There are two potential reasons accounting for this result: (1) The segmentation accuracy of OARs is excellent, resulting in a strong concordance between unedited OARs and the edited OARs, especially for the rectum, bladder, small intestine, and colon. Hence, there is a minor difference in dosimetry between the edited and unedited OARs plans. (2) The dose predictions generated by automatic planning are mainly associated with the OARs of the training data. Even with the input of unedited OARs, diverse differences cannot be predicted. The predicted dose is mostly based on the dose in the training set style, approaching the dose distribution of plans based on edited OARs. What is more, this study only considered unedited OARs, and did not account for the unedited targets. Although the dose differences are not significant, it is necessary to check the organs and make appropriate modifications, especially for patients with relatively large changes [46].

Whether it is dosimetric analysis or clinical evaluation, most automated plans are clinically acceptable. However, for the purpose of automated radiation, the time of auto-plans should be sped up, which will be the future work for us. In addition, more validation of the auto-segmented and auto-planning model should be obtained.

## 5. Conclusions

To the best of our knowledge, this study represents the most comprehensive investigation of targets and OARs in prostate cancer, encompassing both auto-segmentation and auto-planning. The accuracy and efficiency of auto-segmentation meet the requirements for automated radiotherapy in prostate cancer. The dosimetric distribution and clinical evaluation of auto-planning are superior to those of manual planning, though there is still room for improvement in efficiency. The lack of significant dosimetric differences between OAR-edited and OAR-unedited auto-plans suggests that OAR editing can be selectively minimized for distant structures to enhance automated workflow efficiency without compromising treatment quality.

## Figures and Tables

**Table 1:** Treatment planning objectives and dose constraints for the OARs. OARs: organs at risk.

OARs	Parameter	Objective	OARs	Parameter	Objective
Bladder	V70 Gy	<5%	Rectum	V70 Gy	<10%
	V60 Gy	<20%		V60 Gy	<20%
	V50 Gy	<40%		V50 Gy	<40%
	V40 Gy	<50%		V40 Gy	<50%
Small intestine	Dmax	<54 Gy	Colon	Dmax	<60 Gy
	V40 Gy	<100 cc		V40 Gy	<100 cc
	V30 Gy	<250 cc		V30 Gy	<250 cc
Anal canal	Dmax	<70 Gy	Femoral heads	V50 Gy	<5%
	V60 Gy	<10%	Penile bulb	V65 Gy	<30%
	V50 Gy	<20%		V50 Gy	<70%
	V20 Gy	<70%	Pelvic bone	D mean	<32 Gy

**Table 2:** Geometric comparison between auto-segmented contours and manual contours. GTV: gross tumor volume; CTV: clinical tumor volume; *DSC*: Dice similarity coefficient; *HD95%*: 95th-percentile Hausdorff distance.

Structure	*DSC*	*HD95%/mm*	*Recall*	*Precision*	Time/s
GTV	0.87 ± 0.06	4.70 ± 2.56	0.94 ± 0.05	0.82 ± 0.10	7.12 ± 0.51
CTV	0.88 ± 0.12	5.26 ± 4.53	0.92 ± 0.13	0.86 ± 0.08	7.34 ± 0.52
CTVnd	0.82 ± 0.06	16.50 ± 9.75	0.89 ± 0.08	0.77 ± 0.09	7.29 ± 0.56
Rectum	0.89 ± 0.05	6.35 ± 4.18	0.92 ± 0.06	0.86 ± 0.07	6.12 ± 0.56
Small bowel	0.84 ± 0.06	9.52 ± 5.38	0.86 ± 0.10	0.84 ± 0.05	6.23 ± 0.48
Colon	0.83 ± 0.10	15.65 ± 14.96	0.86 ± 0.14	0.80 ± 0.13	6.37 ± 0.49
Bladder	0.96 ± 0.01	3.35 ± 1.56	0.98 ± 0.02	0.94 ± 0.03	6.55 ± 0.56
Anal canal	0.83 ± 0.08	3.69 ± 1.47	0.86 ± 0.08	0.82 ± 0.14	6.11 ± 0.51
Penile bulb	0.65 ± 0.16	3.73 ± 1.74	0.71 ± 0.20	0.66 ± 0.23	6.21 ± 0.51
Pelvic bone	0.91 ± 0.01	5.97 ± 1.81	0.88 ± 0.03	0.94 ± 0.04	6.12 ± 0.46
Femoral Head L	0.89 ± 0.12	7.32 ± 3.61	0.88 ± 0.05	0.93 ± 0.15	6.65 ± 0.52
Femoral Head R	0.91 ± 0.02	7.36 ± 3.28	0.86 ± 0.05	0.96 ± 0.04	6.67 ± 0.53
Mean	0.86	7.45	0.88	0.85	6.61

**Table 3:** Dosimetric differences for PTVs and OARs between auto-plans (edited/manual OARs versus unedited/auto-segmented OARs) and manual plans with manual contours. *HI*: homogeneity index; *CI:* conformity index; PCTV: planning clinical tumor volume; PGTV: planning gross tumor volume; Coverage: the percentage of the target volume covered by the prescribed dose; Vxx Gy (%): the percentage of the organ volume receiving a dose of xx Gy; Dmax/Dmean (Gy): the maximum and mean doses for the organ, respectively; *P1*: statistical difference of manual plans (manual contours) and auto-plans (manual contours); *P2:* statistical difference of auto-plans (manual contours) and auto-plans (unedited OARs). The bold indicates a statistically difference.

Structure	Metric	Auto-Plans (Manual Contours)	Manual Plans (Manual Contours)	Auto-Plans (Unedited OARs)	*P1*	*P2*
PCTVnd	*HI*	0.39 ± 0.07	0.40 ± 0.06	0.37 ± 0.08	0.09	0.59
	*CI*	0.64 ± 0.03	0.55 ± 0.05	0.66 ± 0.05	**0.00**	0.83
	coverage	0.99 ± 0.01	0.98 ± 0.01	0.99 ± 0.01	0.05	0.69
PCTV	*HI*	0.10 ± 0.02	0.09 ± 0.03	0.10 ± 0.01	**0.00**	0.99
	*CI*	0.85 ± 0.15	0.81 ± 0.15	0.87 ± 0.06	**0.01**	0.94
	coverage	0.99 ± 0.01	0.99 ± 0.01	0.99 ± 0.01	0.52	0.76
PGTV	*HI*	0.06 ± 0.01	0.05 ± 0.01	0.06 ± 0.01	**0.02**	0.358
	*CI*	0.72 ± 0.10	0.65 ± 0.12	0.71 ± 0.05	**0.00**	0.27
	coverage	0.99 ± 0.00	0.99 ± 0.01	0.99 ± 0.00	0.91	0.74
Bladder	V30 Gy (%)	60.73 ± 11.05	74.85 ± 13.66	62.80 ± 12.70	**0.00**	0.69
	V40 Gy (%)	40.61 ± 8.40	42.70 ± 7.71	41.57 ± 8.87	**0.02**	0.88
	V50 Gy (%)	19.87 ± 6.16	20.60 ± 6.42	20.36 ± 6.12	**0.04**	0.81
	V55 Gy (%)	14.21 ± 5.37	15.23 ± 5.91	14.01 ± 5.46	**0.00**	0.81
	V60 Gy (%)	10.32 ± 4.20	10.94 ± 4.61	10.22 ± 4.06	**0.00**	0.93
	V65 Gy (%)	6.83 ± 3.00	6.94 ± 3.12	6.85 ± 2.61	0.30	0.86
	Dmax(Gy)	71.45 ± 3.02	70.50 ± 2.96	71.65 ± 3.93	**0.00**	0.50
	Dmean(Gy)	36.46 ± 3.43	39.55 ± 3.05	39.70 ± 13.19	**0.00**	0.96
Rectum	V30 Gy (%)	60.92 ± 10.54	77.73 ± 11.95	62.40 ± 10.24	**0.00**	0.71
	V40 Gy (%)	36.55 ± 7.70	44.25 ± 8.50	38.34 ± 5.63	**0.00**	0.97
	V50 Gy (%)	17.98 ± 4.34	22.59 ± 5.20	18.53 ± 4.53	**0.00**	0.59
	V55 Gy (%)	13.28 ± 4.57	16.32 ± 5.30	13.39 ± 4.94	**0.00**	0.65
	V60 Gy (%)	9.03 ± 3.59	10.91 ± 4.21	9.30 ± 3.74	**0.01**	1.00
	V65 Gy (%)	4.92 ± 2.33	5.09 ± 2.40	5.30 ± 2.46	0.73	0.82
	Dmean (Gy)	36.85 ± 2.54	40.40 ± 2.53	37.28 ± 2.30	**0.00**	0.79
	Dmax (Gy)	70.04 ± 3.45	68.14 ± 3.25	70.26 ± 3.74	**0.00**	0.83
Small Bowel	V30 Gy (cc)	68.38 ± 58.95	116.52 ± 82.71	70.72 ± 63.51	**0.00**	0.94
	V40 Gy (cc)	38.85 ± 38.26	47.74 ± 39.81	39.71 ± 38.79	0.22	0.94
	V50 Gy (cc)	0.45 ± 0.89	0.45 ± 0.90	0.65 ± 1.29	0.87	0.74
	Dmax (Gy)	48.10 ± 10.65	47.67 ± 9.82	46.27 ± 14.48	0.37	0.83
Colons	V30 Gy (cc)	56.51 ± 33.89	79.8 ± 42.6	58.87 ± 32.14	**0.00**	0.85
	V40 Gy (cc)	37.53 ± 27.47	43.6 ± 27.93	37.99 ± 25.22	0.08	0.96
	Dmax (Gy)	52.69 ± 8.72	53.14 ± 3.72	53.58 ± 3.94	0.89	0.32
Anal Canal	V20 Gy (%)	49.43 ± 20.36	57.86 ± 19.25	48.42 ± 22.12	**0.03**	0.45
	V50 Gy (%)	3.70 ± 4.64	4.66 ± 4.82	3.51 ± 2.74	0.41	0.70
	V60 Gy (%)	0.76 ± 1.64	0.84 ± 1.63	0.63 ± 1.13	0.92	0.78
Penile Bulb	V50 Gy (%)	46.38 ± 26.09	48.28 ± 28.06	55.66 ± 31.74	0.72	0.72
	V65 Gy (%)	15.67 ± 21.81	16.05 ± 18.05	20.94 ± 24.22	0.65	0.66
Pelvic Bone	V20 Gy (%)	76.04 ± 6.20	84.49 ± 4.61	76.38 ± 4.84	**0.00**	0.89
	V30 Gy (%)	46.19 ± 8.11	65.77 ± 6.52	47.18 ± 7.90	**0.00**	0.50
	Dmean (Gy)	30.20 ± 2.69	35.13 ± 2.60	30.69 ± 2.69	**0.00**	0.65

## Data Availability

Data are available upon request.

## Abbreviations

OAR	Organ at risk
DSC	Dice similarity coefficient
VR	Volume ratio
HD95%	Hausdorff distance at 95%
VRD	Volume ratio degree
GTV	Gross tumor volume
CTV	Clinical tumor volume
CTVnd	Nodal clinical target volume
PTV	Planning target volume
CI	Conformity index
HI	Homogeneity index
ART	Adaptive radiotherapy
DVH	Dose volume histogram
FBCT	Fan beam CT

## References

[1] Siegel R.L., Giaquinto A.N., Jemal A. (2024). Cancer statistics, 2024. CA Cancer J. Clin..

[2] Bray F., Laversanne M., Sung H., Ferlay J., Siegel R.L., Soerjomataram I., Jemal A. (2024). Global cancer statistics 2022: GLOBOCAN estimates of incidence and mortality worldwide for 36 cancers in 185 countries. CA Cancer J. Clin..

[3] Ma T.M., Neylon J., Casado M., Sharma S., Sheng K., Low D., Yang Y., Steinberg M.L., Lamb J., Cao M. (2022). Dosimetric impact of interfraction prostate and seminal vesicle volume changes and rotation: A post-hoc analysis of a phase III randomized trial of MRI-guided versus CT-guided stereotactic body radiotherapy. Radiother. Oncol..

[4] Alexander S.E., McNair H.A., Oelfke U., Huddart R., Murray J., Pathmanathan A., Patel P., Sritharan K., van As N., Tree A.C. (2022). Prostate Volume Changes during Extreme and Moderately Hypofractionated Magnetic Resonance Image-guided Radiotherapy. Clin. Oncol. (R. Coll. Radiol.).

[5] Zhou S., Luo L., Li J., Lin M., Chen L., Shao J., Lu S., Ma Y., Zhang Y., Chen W. (2019). Analyses of the factors influencing the accuracy of three-dimensional ultrasound in comparison with cone-beam CT in image-guided radiotherapy for prostate cancer with or without pelvic lymph node irradiation. Radiat. Oncol..

[6] Kupelian P.A., Lee C., Langen K.M., Zeidan O.A., Mañon R.R., Willoughby T.R., Meeks S.L. (2008). Evaluation of image-guidance strategies in the treatment of localized prostate cancer. Int. J. Radiat. Oncol. Biol. Phys..

[7] Yan D., Georg D. (2018). Adaptive radiation therapy. Z. Med. Phys..

[8] Yang Y.X., Yang X., Jiang X.B., Lin L., Wang G.-Y., Sun W.-Z., Zhang K., Li H., Jia L.-C., Wei Z.-Q. (2024). Artificial Intelligence-Empowered Multistep Integrated Radiation Therapy Workflow for Nasopharyngeal Carcinoma. Int. J. Radiat. Oncol. Biol. Phys..

[9] Christiansen R.L., Dysager L., Hansen C.R., Jensen H.R., Schytte T., Nyborg C.J., Bertelsen A.S., Agergaard S.N., Mahmood F., Hansen S. (2022). Online adaptive radiotherapy potentially reduces toxicity for high-risk prostate cancer treatment. Radiother. Oncol..

[10] Alongi F., Rigo M., Figlia V., Cuccia F., Giaj-Levra N., Nicosia L., Ricchetti F., Sicignano G., De Simone A., Naccarato S. (2020). 1.5 T MR-guided and daily adapted SBRT for prostate cancer: Feasibility, preliminary clinical tolerability, quality of life and patient-reported outcomes during treatment. Radiat. Oncol..

[11] Leeman J.E., Cagney D.N., Mak R.H., Huynh M.A., Tanguturi S.K., Singer L., Catalano P., Martin N.E., D’Amico A.V., Mouw K.W. (2022). Magnetic Resonance-Guided Prostate Stereotactic Body Radiation Therapy With Daily Online Plan Adaptation: Results of a Prospective Phase 1 Trial and Supplemental Cohort. Adv. Radiat. Oncol..

[12] Qiu Z., Olberg S., den Hertog D., Ajdari A., Bortfeld T., Pursley J. (2023). Online adaptive planning methods for intensity-modulated radiotherapy. Phys. Med. Biol..

[13] Rigaud B., Anderson B.M., Yu Z.H., Gobeli M., Cazoulat G., Söderberg J., Samuelsson E., Lidberg D., Ward C., Taku N. (2021). Automatic Segmentation Using Deep Learning to Enable Online Dose Optimization During Adaptive Radiation Therapy of Cervical Cancer. Int. J. Radiat. Oncol. Biol. Phys..

[14] Huang S., Cheng Z., Lai L., Zheng W., He M., Li J., Zeng T., Huang X., Yang X. (2021). Integrating multiple MRI sequences for pelvic organs segmentation via the attention mechanism. Med. Phys..

[15] Duan J., Bernard M., Downes L., Willows B., Feng X., Mourad W.F., St Clair W., Chen Q. (2022). Evaluating the clinical acceptability of deep learning contours of prostate and organs-at-risk in an automated prostate treatment planning process. Med. Phys..

[16] Hardcastle N., Cook O., Ray X., Moore A., Moore K.L., Pryor D., Rossi A., Foroudi F., Kron T., Siva S. (2021). Personalising treatment plan quality review with knowledge-based planning in the TROG 15.03 trial for stereotactic ablative body radiotherapy in primary kidney cancer. Radiat. Oncol..

[17] Liu C., Gardner S.J., Wen N., Elshaikh M.A., Siddiqui F., Movsas B., Chetty I.J. (2019). Automatic Segmentation of the Prostate on CT Images Using Deep Neural Networks (DNN). Int. J. Radiat. Oncol. Biol. Phys..

[18] Duke S.L., Tan L.T., Jensen N.B.K., Rumpold T., De Leeuw A.A.C., Kirisits C., Lindegaard J.C., Tanderup K., Pötter R.C., Nout R.A. (2020). Implementing an online radiotherapy quality assurance programme with supporting continuous medical education-report from the EMBRACE-II evaluation of cervix cancer IMRT contouring. Radiother. Oncol..

[19] Sun W., Shi Z., Yang X., Huang S., Liao C., Zhang W., Li Y., Huang X. (2024). The performance of a new type accelerator uRT-linac 506c evaluated by a quality assurance automation system. J. Appl. Clin. Med. Phys..

[20] Yu L., Zhao J., Xia F., Zhang Z., Liu Y., Zhang W., Zhou J., Wang J., Hu W., Zhang Z. (2023). Technical note: First implementation of a one-stop solution of radiotherapy with full-workflow automation based on CT-linac combination. Med. Phys..

[21] Peng H., Zhang J., Xu N., Zhou Y., Tan H., Ren T. (2023). Fan beam CT-guided online adaptive external radiotherapy of uterine cervical cancer: A dosimetric evaluation. BMC Cancer.

[22] Huang S., Zhong Z., Pang Y., Zheng W., Liu Y., He M., He L., Yang X. (2023). Validation of bowel and bladder preparation by rectum and bladder variation in prostate radiotherapy based on cone beam CTs. J. Radiat. Res. Appl. Sci..

[23] Ronneberger O., Fischer P., Brox T., Navab N., Hornegger J., Wells W., Frangi A. (2015). U-Net: Convolutional Networks for Biomedical Image Segmentation. Medical Image Computing and Computer-Assisted Intervention–MICCAI 2015. MICCAI 2015.

[24] Paszke A., Gross S., Massa F., Lerer A., Bradbury J., Chanan G., Killeen T., Lin Z., Gimelshein N., Antiga L. (2019). PyTorch: An Imperative Style, High-Performance Deep Learning Library. arXiv.

[25] Isensee F., Jaeger P.F., Kohl S.A.A., Petersen J., Maier-Hein K.H. (2021). nnU-Net: A self-configuring method for deep learning-based biomedical image segmentation. Nat. Methods.

[26] Peng J., Yu L., Xia F., Zhang K., Zhang Z., Wang J., Hu W. (2022). Evaluation of a hybrid automatic planning solution for rectal cancer. Radiat. Oncol..

[27] Kingma D.P., Ba J. (2014). Adam: A Method for Stochastic Optimization. arXiv.

[28] Liang S., Tang F., Huang X., Yang K., Zhong T., Hu R., Liu S., Yuan X., Zhang Y. (2019). Deep-learning-based detection and segmentation of organs at risk in nasopharyngeal carcinoma computed tomographic images for radiotherapy planning. Eur. Radiol..

[29] Hodapp N. (2012). Der ICRU-Report 83: Verordnung, Dokumentation und Kommunikation der fluenzmodulierten Photonenstrahlentherapie (IMRT) [The ICRU Report 83: Prescribing, recording and reporting photon-beam intensity-modulated radiation therapy (IMRT)]. Strahlenther. Onkol..

[30] Wong J., Fong A., McVicar N., Smith S., Giambattista J., Wells D., Kolbeck C., Giambattista J., Gondara L., Alexander A. (2020). Comparing deep learning-based auto-segmentation of organs at risk and clinical target volumes to expert inter-observer variability in radiotherapy planning. Radiother. Oncol..

[31] Shao Y., Gao Y., Wang Q., Yang X., Shen D. (2015). Locally-constrained boundary regression for segmentation of prostate and rectum in the planning CT images. Med. Image Anal..

[32] Macomber M.W., Phillips M., Tarapov I., Jena R., Nori A., Carter D., Folgoc L.L., Criminisi A., Nyflot M.J. (2018). Autosegmentation of prostate anatomy for radiation treatment planning using deep decision forests of radiomic features. Phys. Med. Biol..

[33] Ghavami N., Hu Y., Gibson E., Bonmati E., Emberton M., Moore C.M., Barratt D.C. (2019). Automatic segmentation of prostate MRI using convolutional neural networks: Investigating the impact of network architecture on the accuracy of volume measurement and MRI-ultrasound registration. Med. Image Anal..

[34] Nourzadeh H., Watkins W.T., Ahmed M., Hui C., Schlesinger D., Siebers J.V. (2017). Clinical adequacy assessment of autocontours for prostate IMRT with meaningful endpoints. Med. Phys..

[35] Cha E., Elguindi S., Onochie I., Gorovets D., Deasy J.O., Zelefsky M., Gillespie E.F. (2021). Clinical implementation of deep learning contour autosegmentation for prostate radiotherapy. Radiother. Oncol..

[36] Chen W., Li Y., Dyer B.A., Feng X., Rao S., Benedict S.H., Chen Q., Rong Y. (2020). Deep learning vs. atlas-based models for fast auto-segmentation of the masticatory muscles on head and neck CT images. Radiat. Oncol..

[37] De Kerf G., Claessens M., Raouassi F., Mercier C., Stas D., Ost P., Dirix P., Verellen D. (2023). A geometry and dose-volume based performance monitoring of artificial intelligence models in radiotherapy treatment planning for prostate cancer. Phys. Imaging Radiat. Oncol..

[38] Sibolt P., Andersson L.M., Calmels L., Sjöström D., Bjelkengren U., Geertsen P., Behrens C.F. (2020). Clinical implementation of artificial intelligence-driven cone-beam computed tomography-guided online adaptive radiotherapy in the pelvic region. Phys. Imaging Radiat. Oncol..

[39] Yoon S.W., Lin H., Alonso-Basanta M., Anderson N., Apinorasethkul O., Cooper K., Dong L., Kempsey B., Marcel J., Metz J. (2020). Initial Evaluation of a Novel Cone-Beam CT-Based Semi-Automated Online Adaptive Radiotherapy System for Head and Neck Cancer Treatment-A Timing and Automation Quality Study. Cureus.

[40] Byrne M., Archibald-Heeren B., Hu Y., Teh A., Beserminji R., Cai E., Liu G., Yates A., Rijken J., Collett N. (2022). Varian ethos online adaptive radiotherapy for prostate cancer: Early results of contouring accuracy, treatment plan quality, and treatment time. J. Appl. Clin. Med. Phys..

[41] Bijman R., Sharfo A.W., Rossi L., Breedveld S., Heijmen B. (2021). Pre-clinical validation of a novel system for fully-automated treatment planning. Radiother. Oncol..

[42] Jiang C., Ji T., Qiao Q. (2024). Application and progress of artificial intelligence in radiation therapy dose prediction. Clin. Transl. Radiat. Oncol..

[43] Buschmann M., Sharfo A.W.M., Penninkhof J., Seppenwoolde Y., Goldner G., Georg D., Breedveld S., Heijmen B.J.M. (2018). Automated volumetric modulated arc therapy planning for whole pelvic prostate radiotherapy. Strahlenther. Onkol..

[44] Vaniqui A., Canters R., Vaassen F., Hazelaar C., Lubken I., Kremer K., Wolfs C., van Elmpt W. (2020). Treatment plan quality assessment for radiotherapy of rectal cancer patients using prediction of organ-at-risk dose metrics. Phys. Imaging Radiat. Oncol..

[45] Kalet A.M., Luk S.M.H., Phillips M.H. (2020). Radiation Therapy Quality Assurance Tasks and Tools: The Many Roles of Machine Learning. Med. Phys..

[46] Moazzezi M., Rose B., Kisling K., Moore K.L., Ray X. (2021). Prospects for daily online adaptive radiotherapy via ethos for prostate cancer patients without nodal involvement using unedited CBCT auto-segmentation. J. Appl. Clin. Med. Phys..

